# Fixed Quarterly Dosing of Aflibercept after Loading Doses in Neovascular Age-Related Macular Degeneration in Chinese Eyes

**DOI:** 10.3390/jcm13010145

**Published:** 2023-12-27

**Authors:** Daniel H. T. Wong, Kenneth K. W. Li

**Affiliations:** 1 Department of Ophthalmology, United Christian Hospital, Hospital Authority, Hong Kong, China; 2 Department of Ophthalmology, Tseung Kwan O Hospital, Hospital Authority, Hong Kong, China; 3 Department of Ophthalmology, School of Clinical Medicine, LKS Faculty of Medicine, The University of Hong Kong, Hong Kong, China

**Keywords:** anti-VEGF, macular degeneration, aflibercept, treat and extend, quarterly injections

## Abstract

We aimed to investigate the success rate of planned fixed quarterly aflibercept injections after three loading doses (QDA3L) to achieve stability without recurrence in neovascular age-related macular degeneration (nAMD) at a tertiary eye centre. A retrospective study was conducted over five years (2017–2021) by including all consecutive cases of nAMD treated with three initial aflibercept injections four weeks apart, followed by planned injection appointments every 12 to 16 weeks starting from week 20. The primary endpoint was to determine the proportion of patients who maintained disease inactivity at week 52 and week 104. A total of 40 eyes of 40 patients were included. The overall mean age was 80.8, with a male preponderance. The overall success rate in our study population was 52.9% and 53.6% at week 52 and week 104, respectively. The fovea remained dry at 85.3% at week 52 and 82.1% at week 104, and 85.3% and 85.7% of subjects lost fewer than 15 ETDRS letters at week 52 and week 104, respectively. While this study does not suggest the superiority of this regimen, the success and failure rates obtained in our study can be used in the counselling process for this particular fixed treatment regimen for nAMD.

## 1. Introduction

### 1.1. Anti-Vascular Endothelial Growth Factors and the Global Treatment Burden

Age-related macular degeneration (AMD) accounts for 8.7% of all causes of blindness worldwide and is the most common cause of blindness in developed nations [1]. Its prevalence is likely to increase as a consequence of exponential population ageing [1]. The estimated population suffering from AMD worldwide was 196 million in 2020, projected to increase to 288 million by 2040 [1]. Wet, or neovascular, AMD affects 10–15% of AMD patients and is characterised by macular neovascularisation (MNV), where new immature blood vessels grow towards the outer retina, typically from the underlying choroid, resulting in leakage, fluid accumulation, and haemorrhage [2]. Vascular endothelial growth factors (VEGF), as proangiogenic messengers, play a significant role in neovascularisation [2]. Anti-vascular endothelial growth factors (Anti-VEGF) are important medications regarded as the gold standard used to treat neovascular age-related macular degeneration (nAMD) [3]. One of the main goals of anti-VEGF treatment regimens is to minimise the fluctuations of central subfield thickness of the macula to improve functional outcomes [3,4,5]. Different treatment regimens exist, including the pro re nata (PRN) and the treat and extend (T&E) approaches. The T&E regimen was thought by experts to be more proactive and personalised and is associated with decreased treatment and monitoring burden, better utilisation of clinical resources, and minimisation of the risk of visual loss [3].

Treating macular disease with anti-VEGF has been an enormous burden to ophthalmic services globally. Hong Kong is not an exception. Treatment burden involves frequent clinic visits, travel, emotional burden, caregiver support, appointment booking, visual acuity checks, fundus imaging, financial burden for the patients, and workload for the staff involved in administering the injections [6]. In Hong Kong, anti-VEGF treatment is self-financed, hindering access for the underprivileged. It has been mentioned that most public hospitals in the region are less likely to adopt a T&E regimen directly after the loading phase but are more inclined towards PRN regimen because of limited manpower and resources [7]. With the number of injections administered in the territory exponentially rising in recent years due to the ageing population, there is clearly a need to explore better alternatives to the PRN approach and this forms the basis of our study.

### 1.2. The Planned Quarterly Dosing after Three Loading Doses Regimen (QDA3L) Protocol

**Figure 1:**
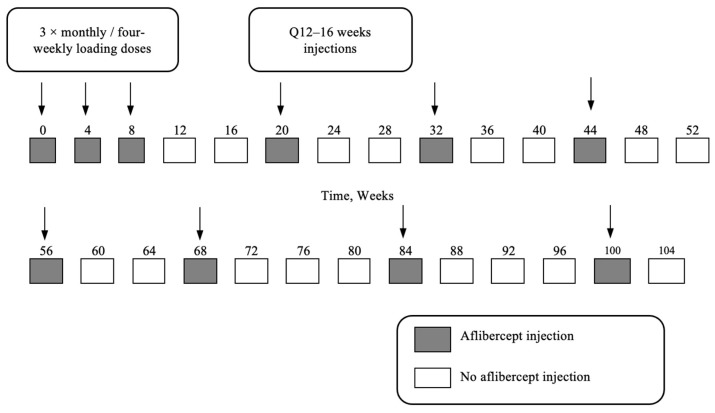
Example of a quarterly dosing after three loading doses (QDA3L) regimen.

Generally, three monthly or four-weekly injections would be arranged for newly diagnosed patients with nAMD during the initial visit at our institution. Due to the difficulty of booking injections well in advance, some ophthalmologists would also help secure a further injection clinic booking at week 20 during that initial visit, on a case-by-case basis, primarily for operational reasons. No specific selection criteria were applied. These patients would be offered a follow-up appointment after the three loading doses, usually at week 12. The objective was to establish a consistent injection interval of 12 to 16 weeks. We have termed this regimen “planned quarterly dosing after three loading doses” (QDA3L) (Figure 1). Previous guidelines suggested that the interval between injections could be extended in four-week increments after the three initial doses, up to a maximum interval of 12 weeks for patients with inactive disease [8]. The ALTAIR study confirmed that a large proportion of patients (35.1–40.5%) had an intended injection interval of 16 weeks by week 52 in patients treated with aflibercept for treatment-naïve exudative AMD [9]. The PIER and EXCITE studies were earlier trials that examined the fixed dosing regimen for ranibizumab [10,11]. The PIER study was a controlled trial that randomized patients with subfoveal MNV to receive sham or ranibizumab monthly for three months, followed by quarterly treatment. The EXCITE study randomised subjects to three initial monthly doses of ranibizumab, followed by a quarterly fixed regimen of different dosing (0.3 mg or 0.5 mg) or monthly ranibizumab. In line with the overall approach of these regimens to ultimately achieve an intended injection interval of 12 to 16 weeks, some of our doctors would discuss with our patients and maintain a fixed quarterly injection interval to sustain disease inactivity. This would reduce the burden of frequent clinic visits when the patient is on a stable treatment protocol.

During each clinic visit, patients underwent visual acuity (VA) testing, optical coherence tomography (OCT), slit lamp biomicroscopy, indirect ophthalmoscopy, and fundus photos for selected cases. During the week 12 appointment and subsequent appointments, the decision on whether to maintain the treatment interval would be made. If there were no signs of increase in activity, the injection at week 20 would be kept, and subsequent injection intervals would be between 12 and 16 weeks (Figure 1). Any decision to shorten the treatment interval to below 12 weeks, due to increased activity, would be regarded as failing the intended planned quarterly dosing.

## 2. Materials and Methods

### 2.1. Study Setting and Population

In light of the increasing treatment burden for all parties involved, we decided to look into whether some fixed, straightforward, and less-treatment intensive regimens would be comparable to the current widely practised treat-and-extend (T&E) regimen [12]. We aimed to investigate the success rate of a planned quarterly aflibercept injection schedule, following three initial loading doses of aflibercept, for neovascular age-related macular degeneration (nAMD) to achieve stability without recurrence at a tertiary eye centre. Our goal is to test the hypothesis that a less frequent dosing regimen may be sustainable to our healthcare system and beneficial to our patients, allowing for a stable disease state without recurrence while reducing the treatment burden and the frequency of visits to an acceptable level.

A retrospective study of electronic health records was conducted, including all consecutive cases of nAMD treated with three initial aflibercept injections four weeks apart, followed by a planned injection appointment at week 20 for at least one year, at United Christian Hospital and Tseung Kwan O Hospital in Hong Kong, over five years (January 2017–December 2021). Our centres provide tertiary eye care to the eastern peninsula of Kowloon in Hong Kong, China, with a catchment population of 1.1 million.

The study received approval from the local research ethics committee (Reference: KC/KE-23-0029/ER-3) and complied with the Declaration of Helsinki.

### 2.2. Inclusion and Exclusion Criteria

Inclusion and exclusion criteria were adopted from major randomized controlled trials (RCTs) [4,9]. The inclusion criteria were as follows: subjects aged over 50 years, neovascular AMD with foveal involvement, both treatment-naïve and recurrent cases, and patients who had injections for at least one year. There were no specific criteria for initial best-correted visual acuity (BCVA). Only active subfoveal MNV were included, including types 1, 2, and 3. Polypoidal choroidal vasculopathy (PCV), a variant of type 1 MNV, was included to represent the real-life data in our locality.

Exclusion criteria were as follows: eyes that received any anti-VEGF therapy in the prior six months, eyes with other disease entities (e.g., diabetic macula oedema, retinal vein occlusions, central serous chorioretinopathy, myopic macular neovascularisation), a follow-up period of less than one year, concurrent macula laser (except photodynamic therapy) and ocular surgery (e.g., cataract surgery or vitrectomy) in the prior six months and the study period, planned injections between week 12 and week 19, and patients who decided to stop injections and opted for a PRN regimen after three injections or within the first year.

### 2.3. Imaging and Evaluation

The spectral-domain optical coherence tomography (SD-OCT) (Spectralis, Heidelberg Engineering, Heidelberg, Germany), fundus fluorescein angiography (FFA), and indocyanine green angiography (ICGA) (Spectralis HRA+OCT, Heidelberg Engineering, Heidelberg, Germany) images were reviewed. In this retrospective study, the neovascular lesions were classified and confirmed via the reviewing of all the multimodal imaging available for each case by one retinal specialist. The subtypes of nAMD were classified according to FFA, ICGA, and OCT findings. Subjects were confirmed to have nAMD with foveal involvement, with confirmation of leakage on fundus fluorescein angiogram before the start of treatment. All cases of PCV were confirmed with ICGA [13]. OCT was the primary imaging modality used to assess dryness of the fovea. Safety evaluation was recorded in clinical notes if any positive findings were observed. Major adverse events screened for included endophthalmitis, retinal detachment, hyphema, vitreous haemorrhage, retinal pigment epithelium tear, thromboembolic events, and death related to the injection.

### 2.4. Treatment Success and Failure

Our definitions of treatment success include overall, anatomical, and functional at W52 and W104. Anatomical success was defined as no increase in disease activity of the AMD on clinical examination or OCT throughout the study period while remaining on the quarterly dosing schedule, without the need to shorten the treatment interval. Functional success was characterised by a loss of fewer than five ETDRS letters, without the need to shorten the treatment interval. Overall success combined both. Treatment failure was defined as an increase in disease activity during the two-year quarterly dosing maintenance stage, i.e. new macular haemorrhage, neovascularisation, and/or an increase in OCT biomarkers for neovascular activity, and thus a decision for rescue treatment with shorter treatment intervals. The widely recognized OCT biomarkers for neovascular activity, including intraretinal fluid, subretinal fluid, subretinal pigment epithelium fluid, and subretinal hyperreflective material, were used when supplementing the clinical findings with OCT response [14,15,16,17].

### 2.5. Statistical Analysis

Frequencies were compared between the groups using either chi-square test or Fisher’s exact test. Continuous unpaired data were compared using Mann–Whitney U test and change in visual acuity with Friedman test and Kruskal–Wallis test. Success rates and outcomes were summarised descriptively. The two-sided 95% confidence intervals of the proportions of success and failure were estimated using normal approximation. A *p* value of less than 0.05 was considered statistically significant. Statistical evaluation was performed using SPSS software version 26 (IBM, Armonk, NY, USA).

## 3. Results

**Figure 2:**
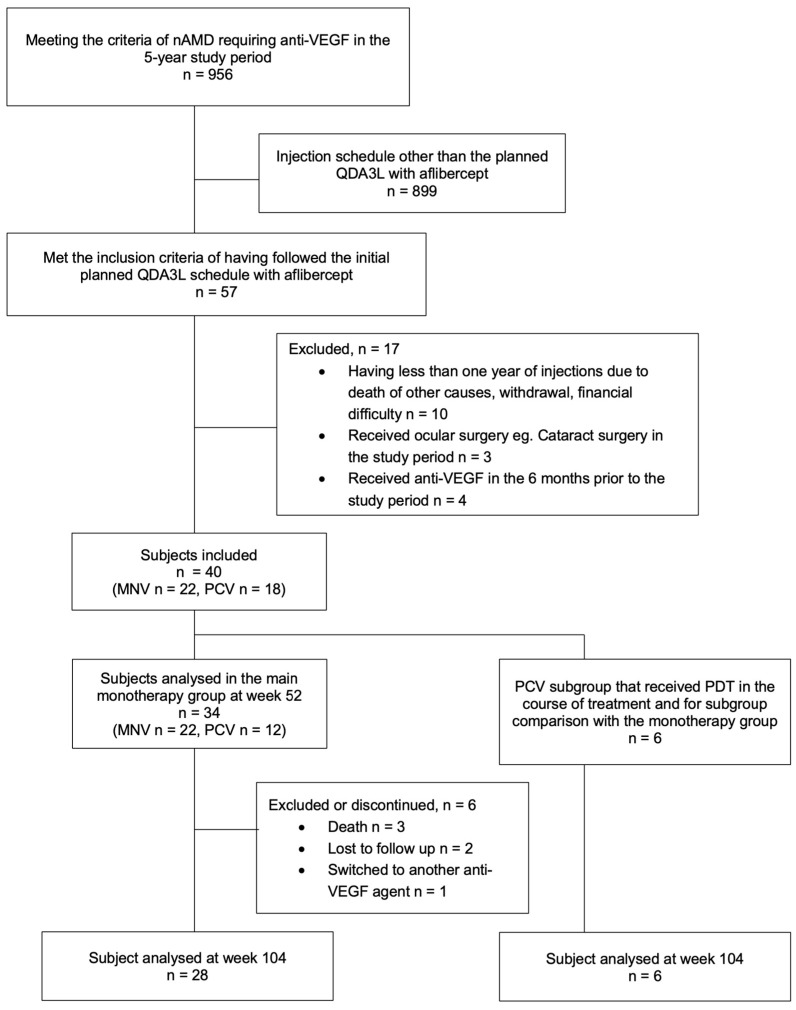
Flow diagram showing the number of patients assessed for eligibility, included in the study, and analysed at each time point. nAMD: neovascular age-related macular degeneration. QDA3L: quarterly dosing after three loading doses. MNV: macular neovascularization. PCV: polypoidal choroidal vasculopathy. PDT: photodynamic therapy. Anti-VEGF: anti-vascular endothelial growth factor.

The flow diagram showing subject recruitment is shown in Figure 2 and the patient demographics are summarised in Table 1. A total of 956 eyes met the criteria of nAMD requiring anti-VEGF in the 5-year study period. Among these, 57 eyes met the inclusion criteria for following the QDA3L schedule with aflibercept. However, 17 of them were excluded due to various reasons, such as not having completed at least one year of injections, voluntary withdrawal, receiving anti-VEGF in the 6 months prior to the study, and undergoing intraocular surgery (Table 2). Of the remaining 40 eyes of 40 patients, 18 cases had polypoidal choroidal vasculopathy (PCV); 6 of them had concurrent photodynamic therapy (PDT) within the first month of their first injection, and thus they were excluded from the main monotherapy group analysis. These six eyes in the combination therapy group were analysed separately. None of the included PCV cases received PDT outside the first month period throughout the study.

A total of 34 eyes of 34 patients were included in the aflibercept monotherapy group and analysed. Among them, 24 cases were treatment-naïve, and 10 were recurrence cases. In total, 64.7% (*n* = 22) of them had a diagnosis of macular neovascularisation (MNV), and 12 of them had PCV with monotherapy. Among the MNV group, 59% (*n* = 13) had type 1 MNV, 36% (*n* = 8) had type 2 MNV, and one case had type 3 MNV.

All patients underwent OCT imaging at their initial visit and subsequent visits. All patients had FFA before their first injection. All PCV cases had ICGA for diagnosis confirmation before their first injection. Not all MNV cases, however, had ICGA: 27% (*n* = 6) only had FFA.

The mean baseline visual acuity was 46.93 ± 14.98 letters. 55.9% (*n* = 19) of the cases were pseudophakic, and the rest had clear lens or mild cataract. A total of 88.2% of the cases did not have any co-existing macula pathology or glaucoma. Four of the cases had mild co-existing pathology such as epiretinal membrane or old retinal vein occlusion. All these did not show any statistical significance in relation to treatment success or failure.

The mean visual acuity across the subjects improved with treatment, and the final visual outcomes are summarised in Table 3. The overall anatomical and functional success at W52 and W104 in our cohort are summarised in Table 4. The mean change in BCVA (ETDRS letters) after three loading doses, from baseline to week 52, and from baseline to week 104 was 5.4 letters (95% CI −0.6 to 11.4), 3.5 letters (95% CI −3.7 to 10.6), and 1.7 letters, respectively (95% CI −4.3 to 7.7). The proportion of subjects who achieved anatomical success at week 52 and week 104 was 73.5% (95% CI 58.7% to 88.4%) and 64.3% (95% CI 46.5% to 82.0%), respectively. The proportion of subjects who achieved functional success at week 52 and week 104 was 55.8% (95% CI 39.2% to 75.6%) and 53.6% (95% CI 35.1% to 72.0%), respectively. The overall success rate in our study population was 52.9% (95% CI 36.2% to 69.7%) at week 52 and 53.6% (95% CI 35.1% to 72.0%) at week 104. The anatomical success rate was higher than the corresponding functional success rate. There were no major adverse events reported during the study period.

Regardless of the anatomical success during the study period, the fovea remained dry on OCT in 85.3% (*n* = 29/34, 95% CI 73.4% to 97.2%) and 82.1% (*n* = 23/28, 95% CI 68.0% to 96.3%) of the cases at week 52 and week 104, respectively. The proportion of subjects who lost fewer than 15 ETDRS letters was 85.3% (95% CI 73.4% to 97.2%) and 85.7% (95% CI 72.8% to 98.7%) at weeks 52 and 104, respectively. The proportion of subjects who gained more than 15 ETDRS letters was 35.3% (95% CI 19.2% to 51.4%) and 25.0% (95% CI 9.0% to 41.0%) at weeks 52 and 104, respectively.

**Figure 3:**
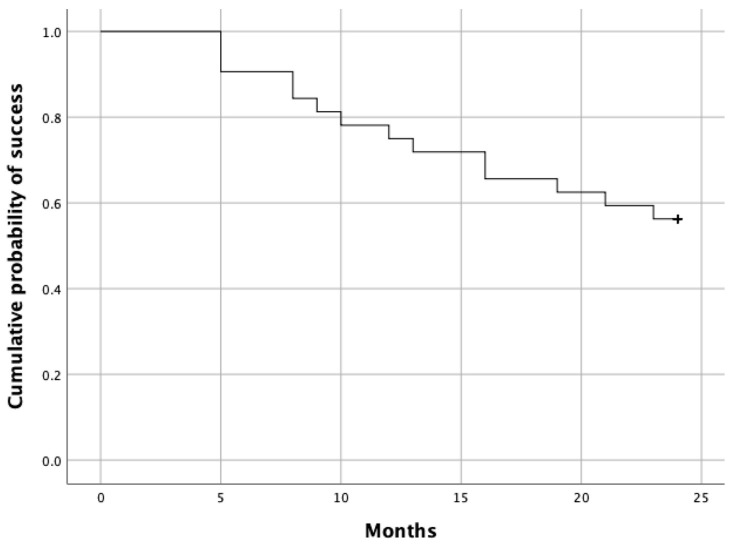
Kaplan–Meier survival curve for treatment to failure time (in months) in our cohort.

The visual acuity after the initial loading doses, but not the VA at weeks 52 or 104, showed a significant difference from with the baseline VA (Friedman test, *p* = 0.01) (Table 3). Between the treatment success and failure groups, there was no statistical difference in the baseline demographics, including gender, age at presentation, baseline visual acuity, whether treatment-naïve or recurrent cases, and lens status. The survival curve is shown in Figure 3. Subgroup analysis for treatment outcomes is shown in Table 5. Statistical analysis did not show a significant difference in outcomes among the different groups of MNV (Table 6). Comparison of outcomes was also performed between the aflibercept monotherapy group and the combination treatment group with PDT for the PCV cases, and the results are shown in Table 7. Due to the small sample size of the PDT subgroup, no significant conclusion can be drawn, although both groups had a fair success rate.

## 4. Discussion

Hong Kong is a densely populated city, and many people in our locality still live in poverty. Although subsidized programmes from the government and charitable programmes from private organisations are available in limited numbers, treating macular disease with anti-VEGF has been an enormous burden to our ophthalmic services. As these injections are locally self-financed, patients who are experiencing financial pressure in our locality cannot afford or are unwilling to comply with tight injection schedules. This group of patients opt out of the treat-and-extend regimen and prefer a pro re nata (PRN) approach instead.

In 2020, a panel of retinal experts from the United Kingdom released practical guidance and recommendations to optimize the aflibercept T&E pathway for nAMD patients that could be implemented in clinical practice [12]. They recommended, for aflibercept, an initial loading phase with three injections four weeks apart, followed by the fourth injection at week 20, and then a decision as to whether to maintain injections every eight weeks or to extend the injection interval by 2 to 4 weeks for active and inactive disease, respectively. The incremental intervals were set at two to four weeks.

Quarterly dosing was studied in larger, multicentre, randomised controlled trials [18]. Both the PIER and EXCITE studies, which were published over a decade ago, studied ranibizumab and found quarterly dosing inferior to monthly dosing [10,11]. There has been a lack of similar studies on fixed quarterly dosing for aflibercept in the past decade. Studies have shown that the intraocular suppression times of VEGF following intravitreal aflibercept in human eyes can last up to 16 weeks [9,19]. Therefore, dosing every 12 to 16 weeks could theoretically be useful to suppress VEGF to prevent recurrence. The subsequent landmark studies, such as ALTAIR, on aflibercept dosing of intervals longer than their labelled bimonthly dosing, were based on a stepwise T&E increment to a 16-week interval [20]. Khanna et al. commented that the switch of research focus to variable frequency anti-VEGF regimens resulted from the unfavourable outcomes from PIER and EXCITE [18]. Subjects in the PIER study were monitored and treated quarterly [18].

Compared to the traditional treat and extend regimen, by planning the fourth injection at week 20, the injection interval will be extended all the way to 12–16 weeks in the QDA3L regimen, without incremental intervals of 2 to 4 weeks. In our study, those who demonstrated increased activity were considered treatment failures and received earlier rescue injections. In prospective real-life practice, we recommend closer monthly follow-up during the initial ‘extend’ period between week 8 and week 20, prior to the injection at week 20. This allows for some degree of individualisation to prevent undertreatment and to identify cases that are stable enough and approaching a dry fovea before transitioning to the QDA3L schedule.

In the ALTAIR study published in 2019, 246 patients were randomised at week 16, after three initial doses of aflibercept, 1:1 to T&E with either 2- or 4-week adjustments [9]. This study was the first randomised controlled study to examine the outcomes associated with 4-week adjustments and injection intervals beyond 12 weeks for aflibercept-treated patients [9]. The proportion of subjects with the last injection interval of 12 weeks or longer was 43.2–49.6% for both groups up to week 52 and 56.9–60.2% up to week 96 [9]. The AIRES study in 2021 was a randomized study involving 271 patients having received aflibercept. Their regimen involved monthly injections till week 16, after which patients were randomised into early-start (with 2-week interval adjustments) or late-start (8-week intervals until W48 then 2-week interval adjustments) T&E [4]. The percentage of patients with the last treatment intervals of 12 weeks or longer up to week 104 was between 47.2 and 51.9% for the early start T&E and late-start T&E groups, respectively [4]. Due to the retrospective nature of our study, while we are not able to compare head-to-head with the randomised trials, our initial study result shows that our protocol has a fair and acceptable response to this group of patients. It should also be noted that our population had a worse baseline visual acuity before treatment and a higher proportion of patients with PCV.

Our study is based on real-life patient data and is not a large randomised clinical trial like ALTAIR or AIRES. In our public healthcare setting, there is a waiting time between the initial visit date and the first injection. Nevertheless, we acknowledge certain weaknesses in the present study, including its retrospective nature and small sample size. The selection of patients into this quarterly regimen by individual doctors at the clinic could result in selection bias, although we did not observe any specific patient population that would be favoured for selection for this booking of the initial four injections at the initial visit, other than for operational reasons. The strict inclusion criteria also mean that this only represented a small proportion of all the injections performed at our institution in the study period. Recent evidence also suggested the use of optical coherence tomography angiography (OCT-A) as a non-invasive technique for detecting macular neovascularisation cases with persistent fluid [14]. We acknowledge the lack of use of such imaging modality in our cases during the study period. Furthermore, it should be noted that our results may not be generalisable to other populations worldwide as all cases in our study were of Chinese ethnicity. Further prospective controlled trials with larger sample size, detailed rescue criteria, and investigations into the success rate of anti-VEGF treatment for different types of MNV may provide additional evidence to support our conclusions.

## 5. Conclusions

Quarterly aflibercept injections after three initial doses were able to maintain a dry fovea for 82.1 to 85.3% of the patients in our locality; 85.3% to 85.7% of patients lost fewer than 15 ETDRS letters, with an overall success rate of 52.9% at 1 year and 53.6% at 2 years. This regimen can be recommended to patients in our locality who have financial constraints and prefer an alternative to the PRN approach, with a lower number of injections. It is important to note that various treatment regimens have been described in the literature, and this retrospective study does not imply the superiority or inferiority of this regimen. Further prospective controlled trials of larger sample sizes may provide more evidence for our conclusions. The success and failure rates observed in our study can provide information for counselling patients considering this specific fixed treatment regimen.

## Figures and Tables

**Table 1:** Patient demographics of the main monotherapy group.

	*n* = 34 (%)	*p* Value for Treatment Success at 1 Year
Age		0.85 #
● Mean ± SD	80.8 ± 7.9	
● Median (min–max)	81 (64–97)	
Laterality, *n* (% left)	13 (38.2%)	0.62 ^
Gender, *n* (% female)	12 (35.2%)	0.47 ^
Ethnicity		
● Chinese	40 (100%)	
Diagnosis		0.22 ^
● MNV	22 (64.7%)	
◦ Type 1 MNV	13	
◦ Type 2 MNV	8	
◦ Type 3 MNV	1	
● PCV	12 (29.4%)	
Baseline visual acuity (ETDRS letters)		0.80 #
● Mean ± SD	46.93 ± 14.98	
● Median (min–max)	46.93 (19.95–73.91)	
Treatment more than 6 months before the first injection in the study period		0.27 ^
● Treatment-naïve	24 (70.5%)	
● Recurrence	10 (29.4%)	
Lens status		0.68 ^
● Pseudophakic	19 (55.9%)	
● Clear lens	1 (0.03%)	
● Nucleus sclerosis grade 1–2	14 (41.1%)	
● Nucleus sclerosis grade 3 or above	0	
Other ocular pathology		
● Nil	30 (88.2%)	-
● Epiretinal membrane	2 (0.05%)	-
● Old branch retinal vein occlusion	1 (0.03%)	-
● Normal tension glaucoma	1 (0.03%)	-

^ Chi-square or Fisher’s exact test. # Mann–Whitney U test. SD: standard deviation. MNV: macular neovascularisation. ETDRS: Early Treatment of Diabetic Retinopathy Study.

**Table 2:** Excluded cases that initially met the quarterly dosing after three loading doses (QDA3L) schedule.

Reasons	*n* = 17
Having less than one year of injections (death of other causes, withdrawal, financial difficulty)	10
Having ocular surgery e.g., cataract surgery in the study period	3
Received anti-VEGF in the 6 months prior to the study period	4

Anti-VEGF: anti-vascular endothelial growth factor.

**Table 3:** Functional outcomes.

	ETDRS Score	*p*-Value #
Baseline visual acuity		
● Mean ± SD	46.93 ± 14.98	-
● Median (min–max)	46.93 (19.95–73.91)	
Visual acuity after first 3 injections		
● Mean ± SD	52.35 ± 17.81	0.01 ^a^ @
● Median (min, max)	58.86 (6.57–80.15)	
Visual acuity at Week 52		
● Mean ± SD	50.37 ± 21.11	0.15 ^a^ 0.26 ^b^
● Median (min, max)	54.45 (19.95–82.71)	
Visual acuity at Week 104 ^		
● Mean ± SD	45.90 ± 22.70	0.47 ^a^ 0.06 ^b^ 0.47 ^c^
● Median (min, max)	46.93 (6.57–77.25)	

# Friedman test with post hoc analysis. ^a^ vs. baseline. ^b^ vs. after three injections. ^c^ vs. week 52. @ *p* < 0.05. ^ Only cases who had follow-up at week 104 (2 years) and who were not excluded from the study were analysed (*n* = 28). ETDRS: Early Treatment of Diabetic Retinopathy Study. SD: Standard deviation.

**Table 4:** Overall, anatomical, and functional success at W52 and W104 in our cohort.

		Anatomical Success #	Functional Success #	Overall Success
Week 52	*n*	25/34	19/34	18/34
	% (95% CI)	73.5% (58.7% to 88.4%)	55.8% (39.2% to 75.6%)	52.9% (36.2% to 69.7%)
Week 104 ^	*n*	18/28	15/28	15/28
	% (95% CI)	64.3% (46.5% to 82.0%)	53.6% (35.1% to 72.0%)	53.6% (35.1% to 72.0%)

# Anatomical success was defined as no increase in activity of the AMD on clinical examination and OCT throughout the study period without the need to shorten the treatment interval. Functional success was characterised by a loss of fewer than five ETDRS letters without the need to shorten the treatment interval. Overall success combined both. ^ 6 were excluded from analysis for functional success at week 104, yielding only 28 cases for analyses, due to the following events after 1 year: death (*n* = 3), lost to follow-up (*n* = 2), switched to another anti-VEGF agent (*n* = 1). 95% CI: 95% confidence interval.

**Table 5:** Subgroup analysis of the overall, anatomical, and functional success at week 52 and week 104 for type 1 and type 2 MNV and PCV monotherapy.

	Anatomical Success	Functional Success	Overall Success
Type 1 MNV—Week 52	10/13 (76.9%)	8/13 (61.5%)	7/13 (53.8%)
Type 2 MNV—Week 52	6/8 (75.0%)	4/8 (50.0%)	4/8 (50.0%)
PCV monotherapy—Week 52	9/12 (75.0%)	7/12 (58.3%)	7/12 (58.3%)
Type 1 MNV—Week 104 ^^a^	5/10 (50.0%)	5/10 (50.0%)	5/10 (50.0%)
Type 2 MNV— Week 104 ^^b^	5/7 (71.4%)	3/7 (42.9%)	3/7 (42.9%)
PCV monotherapy—Week 104 ^^c^	8/11 (72.7%)	7/11 (63.6%)	7/11 (63.6%)

^^a^ Excluded as subjects passed away *n* = 2 and was lost to follow up *n* = 1. ^^b^ Excluded as subject was lost to follow up *n* = 1. ^^c^ Excluded as subject passed away after week 52 *n* = 1. MNV: macular neovascularisation. PCV: polypoidal choroidal vasculopathy.

**Table 6:** Subgroup comparison of outcomes for type 1 MNV, type 2 MNV, and PCV monotherapy (*p*-value).

	After 3 Injections	Week 52	Week 104
Visual acuity gain #	0.49	0.42	0.55
Anatomical success ^	-	0.50	0.87
Functional success ^	-	0.87	0.66
Overall success ^	-	0.93	0.66
Dryness of fovea ^	-	0.86	0.33

# Kruskal–Wallis test. ^ Chi-square test. MNV: macular neovascularisation. PCV: polypoidal choroidal vasculopathy.

**Table 7:** Subgroup analysis of the combo and anti-VEGF monotherapy group of polypoidal choroidal vasculopathy on the overall, anatomical, and functional success at W52 and W104.

	Anatomical Success	Functional Success	Overall Success
Monotherapy—Week 52	9/12 (75.0%)	7/12 (58.3%)	7/12 (58.3%)
Combination therapy—Week 52	5/6 (83.3%)	5/6 (83.3%)	5/6 (83.3%)
Monotherapy—Week 104 ^	8/11 (72.7%)	7/11 (63.6%)	7/11 (63.6%)
Combination therapy—Week 104 ^	4/6 (66.7%)	4/6 (66.7%)	4/6 (66.7%)

^ Excluded at 104 as subject passed away after week 52 *n* = 1. Anti-VEGF: anti-vascular endothelial growth factor.

## Data Availability

The anonymized data presented in this study are available on request from the corresponding author. The data are not publicly available due to patient confidentially.
